# Degradation Acyclovir Using Sodium Hypochlorite: Focus on Byproducts Analysis, Optimal Conditions and Wastewater Application

**DOI:** 10.3390/molecules29163783

**Published:** 2024-08-09

**Authors:** Antonio Medici, Mauro De Nisco, Giovanni Luongo, Giovanni Di Fabio, Marcello Brigante, Armando Zarrelli

**Affiliations:** 1 Department of Chemical Sciences, University of Naples Federico II, 80126 Naples, Italy; antonio.medici@unina.it (A.M.); difabio@unina.it (G.D.F.); 2 Department of Sciences, University of Basilicata, Viale dell’Ateneo Lucano, 85100 Potenza, Italy; mauro.denisco@unibas.it; 3 Associazione Italiana per la Promozione delle Ricerche su Ambiente e Salute Umana, 82030 Dugenta, Italy; giovanni.luongo@unina.it; 4 Institut de Chimie de Clermont-Ferrand, Université Clermont Auvergne, CNRS, Clermont Auvergne INP, F-63000 Clermont-Ferrand, France

**Keywords:** acyclovir, sodium hypochlorite, degradation byproducts, water treatment

## Abstract

In recent years, the environmental impact of pharmaceutical residues has emerged as a pressing global concern, catalyzed by their widespread usage and persistence in aquatic ecosystems. Among these pharmaceuticals, acyclovir (ACV) stands out due to its extensive prescription during medical treatments for herpes simplex virus, chickenpox, and shingles, as well as its heightened usage amidst the COVID-19 pandemic. ACV is excreted largely unchanged by the human body, leading to significant environmental release through wastewater effluents. The urgency of addressing ACV’s environmental impact lies in its potential to persist in water bodies and affect aquatic life. This persistence underscores the critical need for effective degradation strategies that can mitigate its presence in aquatic systems. This study focuses on employing sodium hypochlorite as an oxidative agent for the degradation of ACV, leveraging its common use in wastewater treatment plants. Our research aims to explore the kinetics of ACV degradation, identify and characterize its degradation byproducts, and optimize the conditions under which complete degradation can be achieved. By assessing the efficiency of sodium hypochlorite in real wastewater samples, this study seeks to provide practical insights into mitigating ACV contamination in aquatic environments. The novelty of this research lies in its comprehensive approach to understanding the degradation pathways of ACV and evaluating the feasibility of using sodium hypochlorite as a sustainable solution in wastewater treatment. By addressing the environmental concerns associated with ACV and offering practical solutions, this study contributes to the broader goal of sustainable pharmaceutical waste management and environmental stewardship.

## 1. Introduction

The use of substances for pharmaceutical purposes [1,2,3,4], as disinfectants [5,6,7,8], or clinically for diagnostic purposes [9], for cosmetic products [4,8], or personal hygiene [3,4,8], is extensive and widespread in all areas of daily and professional life. The pharmaceutical industry provides numerous drugs to the community, which are used in human and veterinary medicine for therapeutic, prophylactic, and auxiliary purposes. Pharmaceutical substances, once administered to patients or animals, can be metabolized wholly or partially in the body and eliminated through feces or urine as inactive metabolites, active metabolites, or unchanged substances. Thus, a large quantity of pharmacologically active ingredients is released into the environment, prompting longstanding reflection within the scientific community regarding their fate and environmental impact [10,11,12].

Agricultural practises involving the reuse of solid and liquid manures from livestock farming and sewage sludges to recover nitrogen compounds for soil fertilization may contribute to the spread of drugs in terrestrial environments and, under certain conditions, their entry into water bodies [13,14].

To limit our impact on the environment as much as possible, it is essential to treat domestic and industrial wastewater before it can be discharged into waterways or reused. This role of treating polluted water is performed by wastewater treatment plants (WWTPs), whether internal (as in the case of some industries, where a site may have its own treatment plant) or external (covered or open-air). It is supposed that due to incomplete or completely absent removal processes, with the use of wastewaters, some biologically active substances may be delivered through aqueducts, posing a risk of potential allergic sensitization, especially in infants and the elderly. In several studies, the improvement of organic pollutants degradation using humic substances [15] or physicochemical approaches such as absorption and advanced oxidation processes is proposed for wastewater pollutants removal [16,17,18,19,20,21,22]. Therefore, the ability of removal processes to reduce the presence of substances must be carefully evaluated. Data from the international literature seem to indicate the reassuring suitability of the most common oxidative purification techniques (chemical, physical, and biological, considered individually or in combination with each other) for degrading pharmaceutical substances. However, the use of oxidants in low quantities or in single oxidation cycles [23,24,25,26], as well as the recent proliferation of removal processes (ozonation, UV rays, microfiltration) [27,28,29], all aimed at minimally altering the chemical and sensorial characteristics of the treated water to favour its consumption, could lead to the persistence of pollutants in the effluents of wastewater treatment plants or drinking-water purification plants.

Acyclovir (or aciclovir), often abbreviated as **ACV**, is an antiviral drug capable of interfering with virus DNA synthesis and is primarily used to treat herpes simplex virus infections, chickenpox, and shingles [30]. Other uses include preventing cytomegalovirus infections after transplantation and severe complications of Epstein–Barr virus infection [31]. From a chemical standpoint, Acyclovir is an acyclic analogue of guanosine, one of the nucleosides that make up DNA. Acyclovir is available in numerous medicines administered topically, orally, and intravenously and is one of the most used antiviral drugs [32,33,34,35]. Its consumption has steadily increased since the early 1990s, from around 100,000 kg/p.a. in 1990 to approximately 300,000 just a decade later. It is presumed that consumption further surged during the COVID-19 pandemic [36]. Acyclovir is poorly soluble in water and is characterized by a very short residence time in the human body. High doses are generally prescribed, considering that only 15–20% of the used product is metabolized or accumulated in the human body, while the remaining 80–85% is excreted unchanged through feces and urine [37]. Thus, given its extensive use, it is not surprising to find it in surface waters and effluents from wastewater treatment plants, but its presence in groundwater and even drinking water is certainly concerning. In some industrial discharges, concentrations of up to 2600 mg/L have been found [38], although these are undoubtedly isolated cases linked to criminal management of industrial plants. However, it has been found in effluents from wastewater treatment plants at concentrations of up to 2500 ng/L, as in some Japanese plants [39] or up to 2200 ng/L in Germany [40] and up to almost 2000 ng/L in China [41]. In the United States, it has been detected in surface waters at concentrations of up to 1600 ng/L [42]. It has also been found in groundwater at concentrations of over 130 ng/L [42] and in drinking water at concentrations of up to 40 ng/L [43]. However, these technologies seem unable to achieve complete mineralization of the molecule, and on the other hand, there are few data on any chlorine-based treatments [44]. Indeed, its presence has been measured between 400 and 1360 ng/L in effluents from wastewater treatment plants [42].

The purpose of this study is to investigate the possible fate of Acyclovir when subjected to treatment with sodium hypochlorite, the most used oxidation reaction in wastewater treatment plants. This study aimed to determine the optimal processing conditions to achieve the highest percentage of Acyclovir mineralization and the identification of degradation products. Of these, 11 were identified and quantified, for which a plausible mechanism of formation was proposed.

## 2. Results and Discussion

### 2.1. Degradation Experiments

**Figure 1:**
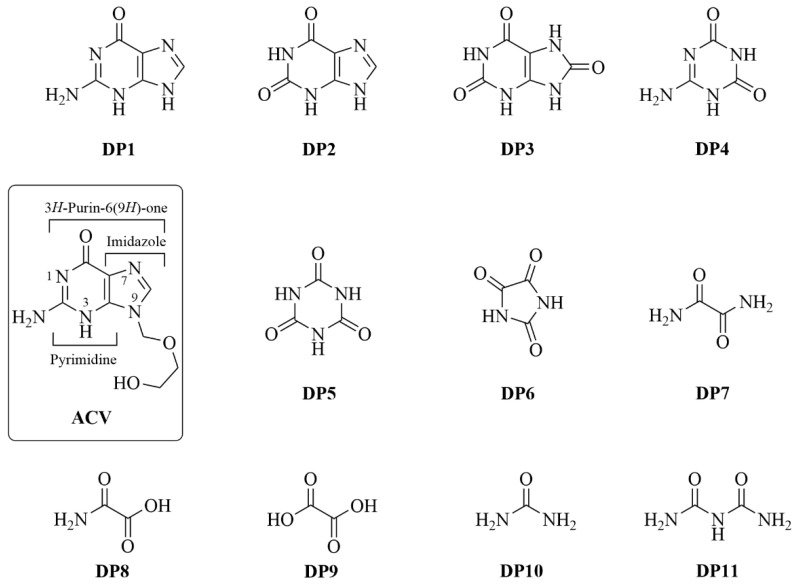
Chemical structures of **ACV** and its degradation products.

The experimental procedures for the oxidation of **ACV** were conducted assuming conditions that ensure the maximum degradation of **ACV**, both in analytical and preparative settings. A solution of **ACV** at a concentration of 10^−5^ M was treated for 20 min in the presence of equimolar hypochlorite at room temperature. Subsequently, experiments were repeated using **ACV** concentrations exceeding 10^−3^ M, employing a significantly higher ratio of hypochlorite (hypochlorite–**ACV**: molar ratio of 7:1) to ensure effective degradation of the target contaminant and to yield adequate quantities of degradation products (**DPs**) for the subsequent structural elucidation. Under these conditions, two experiments were carried out which lasted 20 and 60 min, respectively. The resulting **DPs** (depicted in Figure 1) were purified by column chromatography and HPLC and identified by MS analyses or spectrophotometrically by comparison with commercially available authentic samples.

### 2.2. Structure Elucidation of Degradation Byproducts ***DP1***–***DP11***

In **ACV** treatment, the degradation was monitored by HPLC. The concentration of **DP1**–**DP11** (Figure 1) was at a maximum after 20 min and ranged from 0.3 to 4.9% (Table 1).

Table 1 shows the percentages in which the 11 isolated byproducts were obtained in the two different chlorination reactions.

**Scheme 1:**
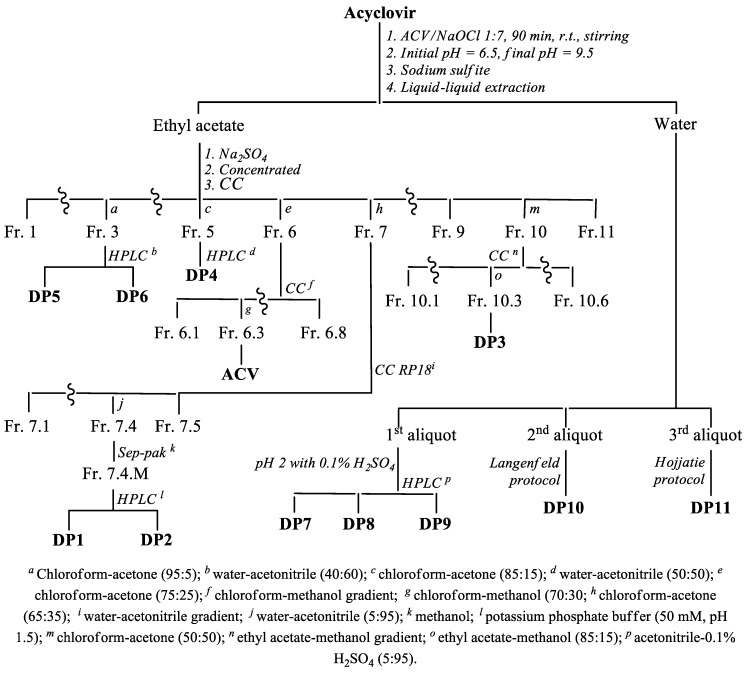
Isolation of the degradation products **DP1**–**DP11**.

**DPs** were isolated by chromatographic processes (Scheme 1) and identified by employing MS analyses or spectrophotometrically by comparison with commercially available authentic samples. These studies often involve the structural determination of the degradation byproducts obtained through one- and two-dimensional NMR analyses. In this case, NMR was not very useful given that the **DPs** obtained were low-molecular-weight compounds, some of which were very soluble in water (**DP7**–**DP11**) and difficult to isolate, and with few diagnostic signals for NMR studies. The use of authentic samples allowed us to identify the **DPs** obtained, quantify them using specific calibration curves, and then further confirm their exact identification using MALDI-TOF mass spectrometry, also working with samples that were not necessarily completely pure.

**Figure 2:**
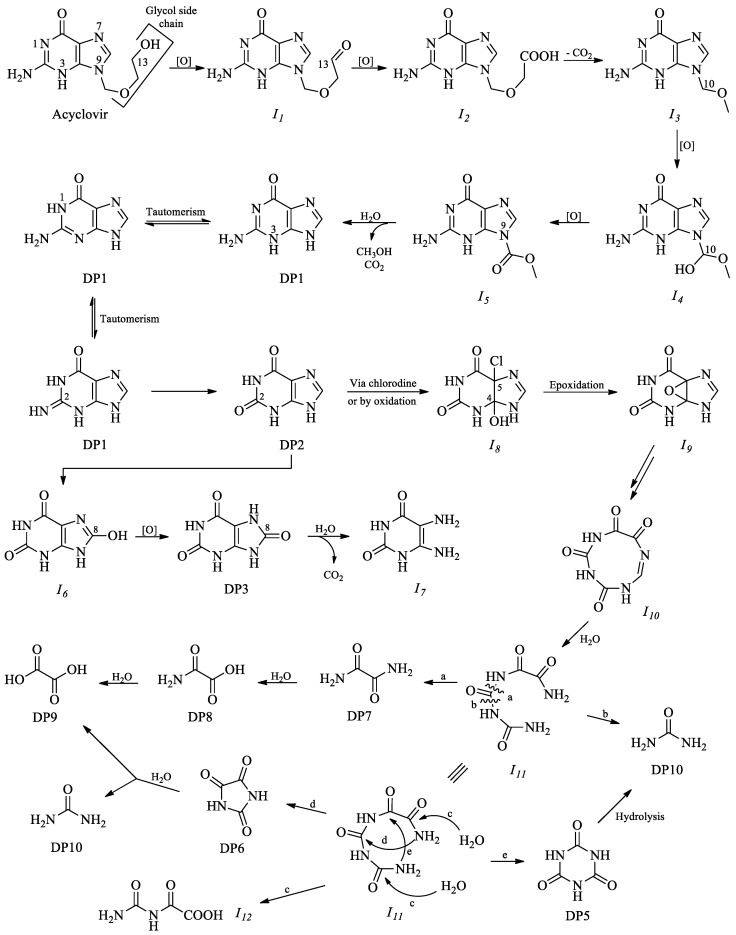
Proposed reaction mechanism leading to the formation of **DP1**–**DP3** and **DP6**–**DP10**. The lowercase letters in the figure indicate possible reaction mechanisms.

**Figure 3:**
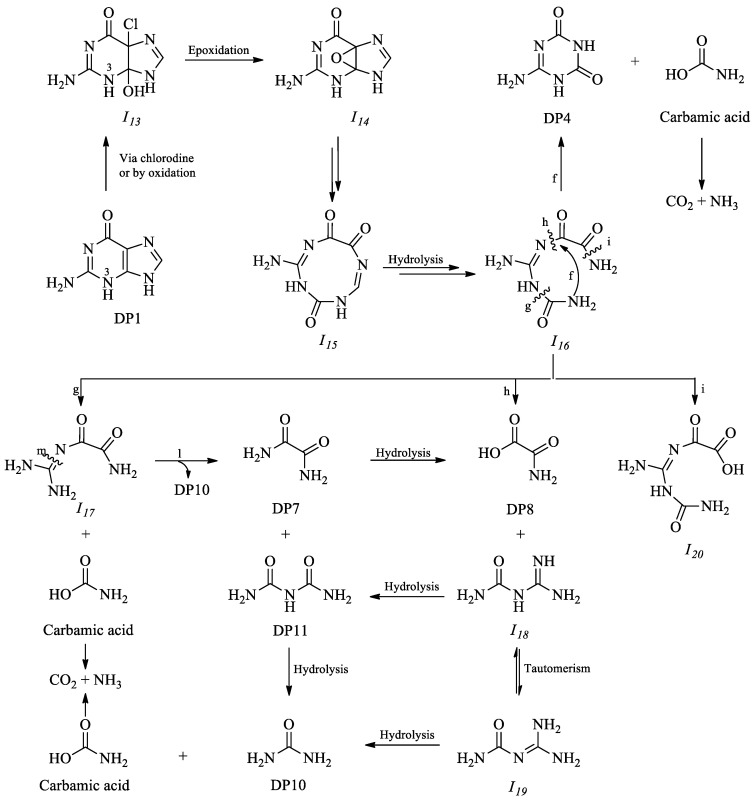
Proposed reaction mechanism leading to the formation of **DP4**, **DP7**–**DP8**, **DP10**–**DP11**. The lowercase letters in the figure indicate possible reaction mechanisms.

Figure 2 and Figure 3 show a plausible mechanism of the **DPs** formation.

In general, given the nature of the isolated and/or identified degradation byproducts, it reasonable to hypothesize that the initial step in the oxidation reaction of **ACV** involves the hydrolysis of its glycol sidechain, resulting in the formation of the degradation byproduct **DP1**.

Indeed, **ACV** could undergo oxidation at the carbon C-13, resulting in the formation of intermediates I_1_ and I_2_, respectively (Figure 2).

Decarboxylation of intermediate I_2_ could yield the intermediate I_3_, which could be oxidized at the carbon C-10 to form the corresponding intermediates I_4_ and I_5_. Hydrolysis of the last intermediate could provide **DP1** as the final product.

The product **DP1**, through tautomerism, could generate its protonated form at the nitrogen N-1 on the pyridine ring and exist in equilibrium with its imine form. The hydrolysis of the latter would lead to the formation of product **DP2**. This byproduct could undergo oxidation at the carbon C-8 of the imidazole ring to form intermediate I_6_, which could then yield, through further oxidation, the product **DP3**. By hydrolysis, the latter could provide the compound *I*_7_, which has not been isolated.

Product **DP2** could also undergo the addition of hypochlorous acid to the double bond between carbons C-4 and C-5, resulting in the formation of intermediate I_8_. Subsequently, through intramolecular epoxidation, intermediate *I*_8_ gives rise to intermediate I_9_. This latter can then undergo hydrolysis and intramolecular oxidation, forming the open intermediate I_11_ through the cyclic intermediate I_10_. Intermediate *I*_11_ may undergo cleavage of an amide bond via two possible pathways: pathway a leads to the formation of product **DP7**, while pathway b results in product **DP10**. **DP7** can subsequently undergo hydrolysis to one of the two amide functions to yield product **DP8**, which then may also undergo hydrolysis at the second amide function to produce **DP9**. Alternatively, intermediate *I*_11_ might undergo an intramolecular attack by one of the two amino functions on one of the carbonyl carbons, as indicated by pathway *c*, to generate compound *I*_12_ (that was not isolated) or by the other amino function, following the pathway d, to form product **DP6**. Hydrolysis of the terminal amide bonds, as indicated by pathway e, leads to the formation of **DP5**.

The **DP1** is a key product that, in the protonated form at the nitrogen N-3 on the pyridine ring, could undergo an oxidation reaction at the double bond between carbons C-4 and C-5, resulting in the formation of intermediate *I*_13_ (Figure 3). This intermediate, through intramolecular epoxidation, could give rise to intermediate *I*_14_, that, through subsequent oxidation and intramolecular hydrolysis, could give rise to intermediate *I*_15_. Upon hydrolysis of the imine function of this last one, intermediate *I*_16_ could be obtained, and the following pathway f, could undergo an intramolecular attack by one of its two amine functions on one carbonyl function, forming **DP4** and carbamic acid. The latter would subsequently degrade to carbon dioxide and ammonia. Hydrolysis of one of the two terminal amide bonds, as indicated by the pathway g, could lead to intermediate *I*_17_ and carbamic acid. From *I*_17_, hydrolysis of the imine bond, as indicated by the pathway l, could yield products **DP7** and **DP10**.

Alternatively, intermediate I_16_ could undergo hydrolysis of its other amide bond, as indicated by pathway h, leading to the formation of product **DP8** and intermediate I_18_. This last one, upon hydrolysis of the imine bond, could create the product **DP11**, while the hydrolysis of the imine bond of its tautomer I_19_ could explain the formation of product **DP10**. **DP10** could also be obtained through the hydrolysis of **DP11**.

Finally, the hydrolysis of the amide bond indicated by pathway i could explain the obtaining of the intermediate I_20_, from which it would be easy to derive many of the isolated products.

With the first chlorination, which lasted 20 min, about one-third or slightly more of the starting product was recovered (Table 1), while the **DP1**–**DP11** products were isolated in a total amount equal to about 30% of the reacted **ACV**. The most abundant product was **DP1** (just over 4%), and the least abundant (less than 0.5%) was **DP11**. It is assumed that the unrecovered **ACV**, about 70% of the initial amount, was either mineralized or transformed into minor products that were not isolated and/or identified.

If the reaction times are tripled under the same experimental conditions, the amount of unrecovered **ACV** decreases to around 4%, and the total amount of **DP1**–**DP11** products accounts for approximatively 19% of the **ACV.** In this case, the most abundant product was **DP10**, with a percentage just below 5%.

### 2.3. Spectral Data and Description of the Isolated Byproducts

The name, physical appearance, chemical formula, theoretical molecular weight, and experimental molecular weight obtained for the pseudo-molecular ion [M + H]^+^ are shown in Table 2.

A total of 11 degradation byproducts have been isolated from Acyclovir; of these, the first 3 (**DP1**−**DP3**) retain the purine ring. In particular, **DP1** is the degradation byproduct deriving from the loss of the methyl glycol side-chain of the starting molecule; **DP2** was devoid of the side-chain and the amino function linked to the carbon C-2 of the pyrimidine ring results oxidized to the corresponding carbonyl group; while, finally, **DP3** is similar to **DP2** but the carbon C-8 of the imidazole ring results also oxidized to the corresponding carbonyl group (Figure 1).

**DP4** and **DP5** products retain only the pyrimidine nucleus of the starting product. Evidently, the latter has undergone the cleavage of bonds C-4/N-9 and C-5/N-7 and oxidation to the carbon C-2 in the case of **DP4** and to the carbons C-2 and C-4 in the case of **DP5**.

**DP6** is actually a derivative of only the imidazole ring of **ACV**, in that all the carbons are oxidized.

The last five products, namely **DP7**−**DP11**, are characterized by a low molecular weight and high water-solubility. Among these, **DP9** can be considered in all respects to be the oxidation product obtained from the glycol side-chain. It is the last product before complete mineralization occurs with the formation of carbon dioxide and water. The byproducts **DP7** and **DP8** are, in principle, the mono- and di-amide derivatives of **DP9**. However, it is easier to imagine that they occurred from the degradation of the aromatic nucleus of **ACV**, as hypothesized in the mechanisms proposed in Figure 2 and Figure 3.

### 2.4. Degration Using Different HOCl:ACV Ratios and in Wastewater

**Figure 4:**
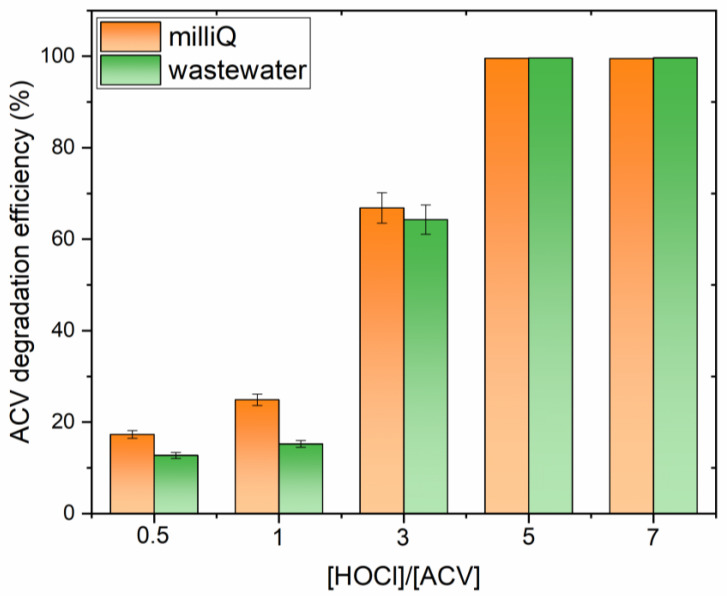
Degradation efficiency of **ACV** after 30 s of reactions in different ratios of hypochlorite–**ACV** in MilliQ and wastewater.

To investigate the degradation of **ACV**, a series of experiments were conducted using a fixed concentration of 10⁻^5^ M **ACV** while varying the concentrations of hypochlorite. In these experiments, 1 mL samples were withdrawn at fixed time intervals and immediately mixed with 10 µL of 0.1 M Na₂S₂O₃ to quench the hypochlorite and prevent further oxidation before analysis [44]. The degradation efficiency of **ACV** after 30 s of reaction, at which point the degradation had reached a plateau, is presented as a function of different hypochlorite–**ACV** ratios in Figure 4. The results indicate a linear increase in **ACV** degradation efficiency from 17% to 98% as the hypochlorite–**ACV** ratio increased from 0.5 to 5. Beyond this ratio, the degradation efficiency reached a plateau, indicating that higher concentrations of hypochlorite did not significantly enhance the degradation process further. This behaviour can be attributed to the saturation point where all available **ACV** molecules have reacted with the hypochlorite. In addition, we explored the impact of using wastewater (Table S1) instead of Milli-Q water to prepare the solutions. It was observed that the degradation efficiency was similar in both cases; however, there was a slight inhibition in the degradation efficiency at lower concentrations of hypochlorite when wastewater was used. This inhibition is likely due to the presence of dissolved organic matter (DOM) in the wastewater, which competes with **ACV** for reaction with hypochlorite. DOM can react with hypochlorite, thus reducing the amount of hypochlorite available to degrade **ACV**. Despite this competitive interaction, complete degradation of **ACV** was still observed when higher concentrations of hypochlorite, specifically five to seven times more, were used.

## 3. Materials and Methods

### 3.1. Drug and Reagents

Acyclovir and sodium hypochlorite solution (6–14% active chlorine) were purchased from Merck (Darmstadt, Germany). Solvents were purchased from Merck (Darmstadt, Germany) and were of HPLC grade and used as received. All other chemicals were of analytical grade and supplied by Merck.

### 3.2. Apparatus and Equipment

Kieselgel 60 (230–400 mesh, Merck, Darmstadt, Germany) was used for column chromatography (CC). HPLC analysis utilized a Shimadzu LC-8A system equipped with a Shimadzu SPD-10A VP UV-VIS detector (Shimadzu, Milan, Italy). MALDI-TOF mass spectrometric analyses were conducted on a Voyager-De Pro MALDI mass spectrometer (PerSeptive Biosystems, Framingham, MA, USA). UV-vis spectra were recorded on a JASCO V-750 UV–Visible Spectrophotometer. Lyophilization of samples was performed using a Lyovapor TM-200 (Buchi, Cornaredo (MI), Italy), with a compressor featuring a cooling capacity of 1.97 kW for 50 Hz and minimum condenser temperature of −55 °C.

### 3.3. Chlorination Reaction

A solution of **ACV** at a concentration of 10^−5^ M was treated with a 10% hypochlorite solution (hypochlorite–**ACV**: molar ratio of 1:1) for 20 min at room temperature. The solution initially had a pH close to neutral, which, after the addition of hypochlorite, reached approximately 8.0. The presence of **ACV** was determined spectrophotometrically with absorbance peaks measured at 252 nm [45] using a previously prepared calibration curve. Under these conditions, the byproducts **DP1**–**DP11** formed were too low in abundance to isolate.

Preparatory experiments were thus conducted, using an **ACV** solution at a concentration higher than 10^−3^ M, treated with a 10% hypochlorite solution. The oxidant was added slowly, dropwise, under magnetic stirring, at room temperature and in a molar ratio approximately seven times higher than that of **ACV** itself. The progress of the reaction was monitored approximately every 10 min by taking aliquots of the solution, which was immediately extracted with ethyl acetate. The organic phase was then analyzed by HPLC. The reaction was stopped after 60 min to ensure maximum degradation of the **ACV** and the formation of its degradation products. The resulting solution was immediately frozen in a bath of acetone and dry ice and then freeze-dried.

The crude reaction was analyzed by HPLC, comparing the chromatographic profile with that obtained from the corresponding experiments under analytical conditions and identifying the common degradation byproducts.

### 3.4. Product Isolation Procedure

Acyclovir (0.85 g, 3.77 mmol) dissolved in MilliQ water (850 mL) was treated with a 10% hypochlorite solution, the concentration of which was determined via iodometric titration (hypochlorite–**ACV**: molar ratio of 7:1) at room temperature. The pH of the solution increased from an initial value of 7.0 to 8.0 within 3 min and remained constant for the duration of the experiment.

Two different chlorination reactions were carried out, with the first stopped after 20 min and the second after 60 min. Immediately after the appointed times, the solutions were frozen at −78 °C and immediately reduced in volume by freeze-drying. Each residue was extracted with ethyl acetate. The ethyl acetate fractions (523 mg after 20 min and 234 mg after 60 min) were chromatographed on silica gel column chromatography (CC) using a gradient of chloroform–acetone to give 11 fractions (Scheme 1). The fraction Fr. 3, eluted with 95:5 chloroform–acetone, was purified by HPLC using a reversed-phase column Phenomenex Synergi 10 μm 110 Å C18 (250 × 10 mm) column and eluted with 40:60 water–acetonitrile to give compounds **DP5** and **DP6**. The fraction Fr. 5, eluted with 85:15 chloroform–acetone, contained compound **DP4**, which was purified by HPLC using the same column as fraction Fr. 3 and eluted with 50:50 water–acetonitrile. The fraction Fr. 6, eluted with 75:25 chloroform–acetone, was rechromatographed on silica gel CC eluted with a gradient of chloroform–methanol, and 8 fractions were obtained. The fraction Fr. 6.3, eluted with 70:30 chloroform–methanol, contained the unreacted **ACV**. The fraction Fr. 7, eluted with 65:35 chloroform–acetone, was chromatographed on reversed-phase silica gel CC with water and acetonitrile, and 5 fractions were obtained. Fraction 7.4, eluted with 5:95 water–acetonitrile, was separated on Sep-Pak RP-18 with methanol and the eluate Fr. 7.4.M was purified by HPLC using a reversed-phase column Phenomenex Luna 5 μm C18(2) (250 × 10 mm), eluted with potassium phosphate buffer (50 mM, pH 1.5), to create **DP1** and **DP2**. The fraction Fr. 10, eluted with 50:50 chloroform–acetone, was rechromatographed on silica gel CC eluted with a gradient of ethyl acetate–methanol, and 6 fractions were obtained. The fraction Fr. 10.3, eluted with 85:15 ethyl acetate–methanol, contained compound **DP3**.

The aqueous phase obtained after the extraction with ethyl acetate was immediately stored in a dark glass bottle with a Teflon cap at 4 °C. An aliquot of this solution was acidified to pH 2 with 0.1% sulfuric acid. The acidified solution was then subjected to chromatographic analysis under ion-exchange conditions using a column Dionex IonPac ICE-AS1 (9 × 250 mm, 7.5 μm particle, Dionex, Sunnyvale, CA, USA). The optimal mobile phase was a mixture of 5:95 acetonitrile–0.1% H_2_SO_4_ at a flow rate of 0.8 mL/min. The sample was prepared in 30:70 acetonitrile–H_2_O with an injection volume of 10 µL and detection was performed at 205 nm. In this way, products **DP7**–**DP9** were identified by comparison with commercial references and quantified by interpolation with their respective calibration curves, obtained using five different solutions of compound **DP7** in concentrations between 2 × 10^−6^ and 7 × 10^−5^ M and five different solutions of **DP8** and **DP9** compounds in concentration between 5.5 × 10^−6^ and 2 × 10^−4^ M.

The qualitative and quantitative determination of urea (**DP10**) was carried out according to a slightly modified version of the protocol described by Langenfeld et al. [46]. Urea was determined by reacting an aliquot of the aqueous solution with diacetyl monoxime in an acidic solution of H_2_SO_4_/H_3_PO_4_ under heat. The resulting species were then complexed with thiosemicarbazide in the presence of ferric chloride. The pink-coloured complex formed has a maximum absorption at 520 nm, which is proportional to the concentration of urea. Its calibration curve was obtained using five different solutions in concentrations between 1 × 10^−3^ and 5 × 10^−3^ M.

A further aliquot of the aqueous phase (50 mL) was lyophilized and then re-dissolved in MilliQ water. The fraction was analyzed via HPLC using a Luna NH_2_ 5 μm column (250 × 4.6 mm, Phenomenex, Bologna, Italy), with an eluent consisting of a solution of 85:15 acetonitrile–water. Detection was performed at a wavelength of 195 nm using a UV detector [47]. The product **DP11** was identified by comparison with a commercial reference and quantified by interpolation using the appropriate calibration curve, obtained using five different solutions in concentrations between 9.7 × 10^−6^ and 4.4 × 10^−5^ M.

## 4. Conclusions

In conclusion, our study highlights the transformations of **ACV** because of chlorination, the most used oxidation method in wastewater treatment systems. **ACV** is an emerging microcontaminant, present even in surface waters and, in some cases, even in drinking water, with the suspicion that its presence, given its widespread use (especially during the COVID-19 pandemic), may constantly increase over the coming years. The chlorination reaction could prove to be a convenient method for treating wastewater to eliminate this pollutant and others that are structurally similar. In fact, while 11 degradation products were isolated, mostly identified through mass spectrometry studies or by comparison with commercially available products, the presence of unaltered recovered Acyclovir decreased from 34% to just 4% by extending the reaction times from 20 to 60 min. The degradation products were obtained in percentages ranging from 0.3% to 4.9%, with a transformation percentage of all isolated byproducts of about 29% for a 20 min chlorination and 19% for a 60 min reaction. A plausible mechanism for their formation has been proposed in relation to the isolated products. Finally, experiments using different ratios of hypochlorite in MilliQ and wastewater highlight the relevance of this work.

## Figures and Tables

**Table 1:** Recovery percentages of Acyclovir and those of its isolated byproducts.

DP	1st Chlorination % *	2nd Chlorination % *
**ACV**	~34	~4
**1**	4.2	0.9
**2**	3.8	1.5
**3**	2.5	1.0
**4**	2.4	0.5
**5**	2.3	1.1
**6**	2.1	0.6
**7**	3.1	3.3
**8**	2.8	3.3
**9**	1.6	2.1
**10**	3.5	4.9
**11**	0.5	0.3
Transformation Σ%**DPs**	≥29	≥19

* The percentages were calculated based on the quantity of ACV that was not recovered unchanged.

**Table 2:** Spectral data of **ACV** and its degradation byproducts **DP1**–**DP11**.

DP	Name	Physical Aspect	Chemical Formula	*m*/*z* Calculated [M]^+^	*m*/*z* Found [M + H]^+^
**ACV**	2-Amino-9-((2-hydro xyethoxy)me thyl)-3H-purin-6(9H)-one	Grey powder	C_8_H_11_N_5_O_3_	225.20	226.08 (88%)
**DP1**	2-Amino-3H- purin-6(9H)-one	Grey powder	C_5_H_5_N_5_O	151.05	152.12 (55%)
**DP2**	1H-Purine- 2,6(3H,9H)-dione.	Grey powder	C_5_H_4_N_4_O_2_	152.03	153.13 (66%)
**DP3**	1H-Purine-2,6,8(3H,7H,9H)-trione	Grey powder	C_5_H_4_N_4_O_3_	168.03	169.12 (38%)
**DP4**	6-Amino-1,3,5-triazine-2,4(1H,3H)-dione	White powder	C_3_H_4_N_4_O_2_	128.03	129.09 (41%)
**DP5**	1,3,5-Triazinane- 2,4,6-trione	White powder	C_3_H_3_N_3_O_3_	129.02	130.07 (42%)
**DP6**	Imidazolidine- 2,4,5-trione	White powder	C_3_H_2_N_2_O_3_	114.01	115.06 (51%)
**DP7**	Oxalamide	Grey powder	C_2_H_4_N_2_O_2_	-	-
**DP8**	2-Amino-2-oxoacetic acid	Grey powder	C_2_H_3_NO_3_	-	-
**DP9**	Oxalic acid	Grey powder	C_2_H_2_O_4_	-	-
**DP10**	Urea	Grey powder	CH_4_N_2_O	-	-
**DP11**	Carbamylurea	Grey powder	C_2_H_5_N_3_O_2_	-	-

## Data Availability

Data are contained within the article.

## Supplementary Materials

The following supporting information can be downloaded at https://www.mdpi.com/article/10.3390/molecules29163783/s1. Table S1. Wastewater physico-chemical characteristics.
