# Supplementary material for: Degradation Acyclovir Using Sodium Hypochlorite: Focus on Byproducts Analysis, Optimal Conditions and Wastewater Application
Source: Molecules. 2024 Aug 9;29(16):3783. doi: 10.3390/molecules29163783 (PMC11357095; doi:10.3390/molecules29163783)
Supplement: Supplementary file 1 [file molecules-29-03783-s001.zip › molecules-3103181-supplementary.pdf]

Degradation Acyclovir using sodium hypochlorite. Focus on byproducts analysis, optimal conditions and wastewater application

Antonio Medici [1], Mauro De Nisco [2], Giovanni Luongo [3], Giovanni Di Fabio [1], Marcello Brigante [4,*] and Armando Zarrelli [1,*]

Table SM1. wastewater physico-chemical caracteristics.

Parameters	STPW
pH	7.2
UV_{254}	0.065
IC (mg_C L^{-1})	64
TOC (mg_C L^{-1})	2.5
Cl^- (mg L^{-1})	94
SO_4^{2-} (mg L^{-1})	45
NO_3^- (mg L^{-1})	50
